# Gift Card Incentives and Non-Response Bias in a Survey of Vaccine Providers: The Role of Geographic and Demographic Factors

**DOI:** 10.1371/journal.pone.0028108

**Published:** 2011-11-23

**Authors:** Joshua Van Otterloo, Jennifer L. Richards, Katherine Seib, Paul Weiss, Saad B. Omer

**Affiliations:** 1 Emory University Preparedness and Emergency Response Research Center, Atlanta, Georgia, United States of America; 2 Hubert Department of Global Health, Rollins School of Public Health, Emory University, Atlanta, Georgia, United States of America; Bremen Institute of Preventive Research and Social Medicine, Germany

## Abstract

This study investigates the effects of non-response bias in a 2010 postal survey assessing experiences with H1N1 influenza vaccine administration among a diverse sample of providers (N = 765) in Washington state. Though we garnered a high response rate (80.9%) by using evidence-based survey design elements, including intensive follow-up and a gift card incentive from Target, non-response bias could exist if there were differences between respondents and non-respondents. We investigated differences between the two groups for seven variables: road distance to the nearest Target store, practice type, previous administration of vaccines, region, urbanicity, size of practice, and Vaccines for Children (VFC) program enrollment. We also examined the effect of non-response bias on survey estimates. Statistically significant differences between respondents and non-respondents were found for four variables: miles to the nearest Target store, type of medical practice, whether the practice routinely administered additional vaccines besides H1N1, and urbanicity. Practices were more likely to respond if they were from a small town or rural area (OR = 7.68, 95% CI = 1.44−40.88), were a non-traditional vaccine provider type (OR = 2.08, 95% CI = 1.06−4.08) or a pediatric provider type (OR = 4.03, 95% CI = 1.36−11.96), or administered additional vaccines besides H1N1 (OR = 1.80, 95% CI = 1.03−3.15). Of particular interest, for each ten mile increase in road distance from the nearest Target store, the likelihood of provider response decreased (OR = 0.73, 95% CI = 0.60−0.89). Of those variables associated with response, only small town or rural practice location was associated with a survey estimate of interest, suggesting that non-response bias had a minimal effect on survey estimates. These findings show that gift card incentives alongside survey design elements and follow-up can achieve high response rates. However, there is evidence that practices farther from the nearest place to redeem gift cards may be less likely to respond to the survey.

## Introduction

Understanding the experiences of physicians and health practitioners is vital to planning for and understanding public health interventions. Many physician surveys are conducted by mail. In a 1991 meta-analysis of 178 articles published in 111 different journals, response rates to mailed physician surveys varied from approximately 20% to 90%, with an average response rate of 54%. In contrast, mail surveys of non-physicians in this meta-analysis had an average response rate of 68% [1]. When survey response rates are low, the study sample may not adequately represent the target population, especially when non-respondents differ from respondents in important ways.

Various methods to increase mail survey response rates have been explored. Specific survey protocol elements have been used successfully to increase response rates, including: cash incentives, inclusion of contact information of many study investigators, personalization and first-class stamps on return envelopes [2], multiple follow-ups, the inclusion of replacement questionnaires during follow-up, the use of short questionnaires [3], and the use of a courier service such as FedEx [4]. Non-cash incentives such as pens, stickers, token donations to charity, entry into a lottery, and informational material have been found to be less effective than cash incentives [5]. Additionally, cash incentives have been shown to increase response rates more than other methods, especially when incentives are upfront rather than promised [6]. Use of gift cards as an alternative monetary incentive has been shown to be more effective at increasing response rates than non-monetary incentives [7].

In previous studies of physician non-response bias, demographic differences were found between respondents and non-respondents, even when high response rates were achieved [8]–[10]. However, the effect of non-response bias on survey measures was negligible or small [9]–[11]. One explanation for this finding was that these studies examined relatively homogenous populations of physicians (e.g., dentists [8], pediatricians [12], or general practitioners [11]).

We conducted a survey of Washington vaccine providers in September–November 2010 to assess vaccine providers' experiences during the H1N1 pandemic. We used an evidence-based protocol which included a gift card incentive. Gift cards allow individual receipt and usage tracking and replacement of lost or stolen cards, reducing risks associated with sending cash by mail. Some of the vaccine providers faced potential barriers to easy use of gift cards, such as long travel distances to the nearest store or lack of internet access for online use of gift cards. Thus, the perceived value of the incentive could be different based upon geographic and demographic factors, which could introduce non-response bias. Additionally, it has been shown that it is more difficult to get high response rates from some vaccine provider types than others, particularly pharmacies [13] and correctional facilities [14].

Since there was significant heterogeneity in characteristics of healthcare providers in Washington, we assessed whether use of gift card incentives introduced non-response bias. Previous studies addressing non-response bias have examined demographic and geographic factors. However, there are no studies addressing the impact of practice type and distance to the nearest location where respondents can redeem incentives in the context of gift card incentives.

The current study investigates demographic and geographic non-response bias in a survey of Washington H1N1 influenza vaccine providers using gift card incentives.

## Methods

We conducted a survey of Washington vaccine providers to investigate experiences, concerns, and use of immunization information systems (IIS) during the 2009–2010 H1N1 influenza immunization campaign. The purpose of the survey was to assess provider response to novel pandemic influenza A, the challenges associated with vaccine priority groups, and the potential to leverage existing systems in vaccine and non-vaccine related emergencies. Due to the liability of sending cash through the mail, we used gift card incentives in lieu of cash in an effort to maximize response rates.

### Ethics

The Emory University Institutional Review Board (IRB) approved the study as exempt (#0004491). The Washington State IRB approved the study as non-human subject research (#E-072110-H). Informed consent was obtained via courier delivery of a Frequently Asked Questions (FAQ) document included with the survey which addressed the purpose, risks and benefits, confidentiality, incentives, and voluntary nature of the survey.

### Sample

We drew a stratified random sample of 800 vaccine providers from 2,523 eligible practices who ordered H1N1 vaccine from the Washington State Department of Health and Human Services during the 2009–2010 H1N1 influenza pandemic. The sample size of 800 was based on a minimum anticipated response rate of 50%, and we sought survey estimates accurate within ±5% for all measures. All women's health providers (n = 107, Table 1) and correctional facilities (n = 31, Table 1) were selected, and the remaining providers were selected by stratified random sample. Women's health providers and correctional facilities were oversampled for pooled analysis with surveys in other states. The remaining six categories of provider types were proportionally represented in the sample: non-traditional vaccinators (e.g., alternative medicine, rehabilitation, occupational health, specialists), under-25-year-old priority group practices (e.g., pediatrics, college health services), pharmacies, government providers (e.g., Indian Health Service, local health jurisdictions, Veterans Affairs), hospitals and acute care, and traditional family practices. After eliminating 34 duplicate addresses and 1 Oregon address, 765 questionnaires were delivered.

**Table 1:** Demographic characteristics by response status and response time.

		Mean (SD) or N(%)*						Group P-Value**	Mean (SD) or N (%)*				Group P-Value**
Variable		Total Sample (n = 765)		Respondent (n = 594)		Non-Respondent (n = 171)			Early Respondent (n = 404)		Late Respondent (n = 180)		
Mean distance to Nearest Target (miles)		12.0	(19.1)	11.9	(19.0)	12.5	(19.4)	0.730	11.3	(17.9)	13.4	(21.6)	0.215
Mean time to nearest Target (minutes)		18.5	(22.5)	18.4	(22.7)	18.5	(22.0)	0.955	17.6	(21.0)	20.3	(26.2)	0.196
Type of Practice (%) ***								0.037					0.063
	Non-Traditional Vaccinators	149	(19.5)	118	(79.2)	31	(20.8)		80	(69.6)	35	(30.4)	
	Pediatric Providers	48	(6.3)	44	(91.7)***	4	(8.3)		34	(77.3)	10	(22.7)	
	Pharmacy Providers	147	(19.2)	102	(69.4)	45	(30.6)		56	(56.0)***	44	(44.0)	
	Government Providers	60	(7.8)	51	(85.0)	9	(15.0)		35	(70.0)	15	(30.0)	
	Hospital Providers	31	(4.1)	26	(83.9)	5	(16.1)		17	(70.8)	7	(29.2)	
	Traditional Family Providers	192	(25.1)	144	(75.0)	48	(25.0)		104	(72.2)	40	(27.8)	
	Corrections Facilities	31	(4.1)	25	(80.6)	6	(19.4)		15	(60.0)	10	(40.0)	
	Women's Health Providers	107	(14.0)	84	(78.5)	23	(21.5)		63	(76.8)	19	(23.2)	
Type of Vaccinator (%) ***								0.013					0.014
	Vaccinator for more than H1N1	296	(38.7)	244	(82.4)***	52	(17.6)		182	(74.9)***	61	(25.1)	
	Vaccinator for only H1N1	469	(61.3)	350	(74.6)	119	(25.4)		222	(65.1)	119	(34.9)	
Region of Washington (%)								0.403					0.429
	North	96	(12.6)	75	(78.1)	21	(21.9)		48	(64.9)	26	(35.1)	
	Northwest	47	(6.1)	39	(83.0)	8	(17.0)		27	(71.1)	11	(29.0)	
	West	81	(10.6)	63	(77.8)	18	(22.2)		39	(62.9)	23	(37.1)	
	Southwest	52	(6.8)	42	(80.8)	10	(19.2)		32	(78.1)	9	(22.0)	
	Tacoma	109	(14.3)	80	(73.4)	29	(26.6)		61	(76.3)	19	(23.8)	
	Seattle	212	(27.1)	161	(75.9)	51	(24.1)		111	(71.6)	44	(28.4)	
	North Central	30	(3.9)	23	(76.7)	7	(23.3)		14	(60.9)	9	(39.1)	
	South Central	64	(8.4)	57	(89.1)	7	(10.9)		35	(61.4)	22	(38.6)	
	East	74	(9.7)	54	(73.0)	20	(27.0)		37	(68.5)	17	(31.5)	
Metro Type (%)								0.680					0.146
	Metropolitan	643	(84.1)	495	(77.0)	148	(23.0)		340	(70.0)	146	(30.0)	
	Micropolitan	81	(10.6)	65	(80.2)	16	(19.8)		41	(64.1)	23	(35.9)	
	Small Town or Rural	41	(5.4)	34	(82.9)	7	(17.1)		23	(67.6)	11	(32.4)	
VFC Status (%)													0.075
	VFC Provider	-		-		-			184	(74.2)	64	(25.8)	
	Non-VFC Provider	-		-		-			203	(67.2)	99	(32.8)	
Mean Daily Number of Patients Seen		-		-		-			49.5	(71.1)	55.4	(111.8)	0.441

Note: total n is not the same for VFC status and mean daily number of patients due to item specific non-response.

*Means and standard deviations are given for continuous variables, counts and percents for categorical variables.

**P-Values reported in this column are group tests. For example, the P-Value reported for type of practice compares the model including practice type variables to the one not including practice type variables by likelihood ratio tests.

***P<0.05. Individually significant variables marked with *** were compared to a reference category by Fisher exact test.

### Materials

Identical printed and online survey instruments were used to collect data from study participants. The printed survey instrument was a five-page, single-sided questionnaire. The paper survey was single-sided in order to facilitate the option of returning the survey by fax. The questionnaire consisted of 39 questions divided into 5 sections: practice demographics (5 items), communication with public health and the public (6 items), 2009 H1N1 vaccination administration (15 items), staff participation in public health preparedness activities (4 items), and use of IIS (8 items). We collected information on practice demographics including questions about provider type, participation in Vaccines for Children (VFC, a federal vaccine program), role of the contact (i.e., the onsite vaccine coordinator to whom the survey was targeted) in the practice, and size of the practice. The communications section of the survey addressed sources of public health information, effective communication methods from public health agencies, and effectiveness of previous local and state public health department communications. Questions addressing vaccine administration covered topics of priority group guidelines, staff vaccine coverage, and challenges of vaccine administration. We asked questions covering preparedness activities including questions about past participation in training or preparedness drills and past involvement in actual emergency responses. Finally, a section on the use of IIS included questions about the use and ease of use of Washington's IIS, Child Profile.

On September 15, 2010, sampled providers received a fax that informed them about the upcoming survey and outlined the survey goals. Two weeks later, we sent the survey by FedEx to study participants as a “survey kit”. Each was addressed to the person identified by the Washington State Department of Health and Human Services as the primary contact for ordering H1N1 vaccine at the practice. We used FedEx for delivery with the goals of increasing response rates and tracking signed receipt of the survey and gift card. Also included in the survey kit were a hard copy of the survey instrument, a cover letter, an informed consent framed as a FAQ page, a postage-paid addressed return envelope, a pen, and a $25 gift card to Target to thank the contact for their time. Target is the second largest discount retailer in the United States [15]. Target stores sell household items, apparel, electronics, and health and grocery products. We chose this retailer for the incentive because it offers a wide selection of merchandise, good geographic coverage, gift cards that are redeemable online, and the ability to delay gift card activation to protect our investment. The cover letter described the contents of the survey kit and the objectives of the survey, provided contact information of the investigators, and indicated ways that respondents could complete the survey (mail, fax, or online). In addition to addressing general concerns about confidentiality, the voluntary nature of the survey and the risks and benefits of the survey, the FAQ addressed gift card use, survey funding, and the multiple ways to return the survey. The website address of the online survey tool was chosen to be simple and was printed on all survey materials. Gift cards could be used in-store at any Target location or could be used online at Target.com. The online survey tool was administered using Feedback Server version 2008.1 (Geneva, Switzerland).

Non-respondents received a fax reminder two weeks after the first mailing, including the full survey instrument, cover letter, and survey FAQ document. Three weeks after the first mailing, we contacted non-respondents by telephone a maximum of three times over a period of 9 weeks. We left voicemail messages with the provider contact if direct contact was not possible after the first follow-up. Missing, incomplete, or outdated information was updated during telephone follow-up with the vaccine provider. Nine weeks after the initial mailing, remaining non-respondents received a personalized fax reminder. The reminder included the full survey instrument, a history of follow-up with that individual to date, and a reminder that his/her response was valuable for obtaining a representative sample.

### Measures

We assessed non-response bias by comparing survey respondents with non-respondents, and by comparing early respondents to late respondents by demographic and practice-related variables: road distance in miles or in minutes to the nearest Target store, type of practice, geographic region of Washington, degree of urbanicity (as defined by the U.S. Census Bureau), whether the practice regularly administers vaccines, and size of practice. Each of these predictors has either been shown previously to affect response rates or was of particular interest in this study.

We used GoogleMaps to calculate road distance in miles and driving time needed to reach the nearest Target store from each provider by the shortest possible route. Late response was defined as responding after the first fax follow-up two weeks after delivery, and early response was defined as responding before the first follow-up. Demographic and practice-level data about providers was obtained from the Washington State Department of Health and Human Services, including physical address, local health jurisdiction, and whether the practice had registered to provide vaccines other than H1N1. We categorized geographic regions in Washington using region categories defined by the Washington State Department of Health and Human Services [16]. Respondents self-reported provider category (type of practice) and size of practice. Degree of urbanicity was determined using Rural-Urban Commuting Area (RUCA) codes obtained by ZIP code approximation through the Rural Health and Research Center [17]. We combined small town and rural designations to permit analysis due to small strata sizes.

The three survey estimates of interest were ease of adherence to priority group guidelines, perceived capability to respond to future public health emergencies, and participation in training drills or emergency preparedness exercises. We dichotomized ease of adherence to priority group guidelines into those responding “Easy - The guidelines made it easy for our practice to make decisions on who should or should not receive the vaccine.” compared to those responding “Moderate -The guidelines gave us general guidance, but we still had to make some case-by-case decisions that we were not sure were covered by the guidelines.” or “Hard - In most cases, the guidelines were not specific enough to help our practice make decisions on who should receive vaccine.” We categorized perceived capability to respond to future public health emergencies into those practices responding “Strongly Agree” or “Agree”, compared to all other responses on a five-point Likert Scale from “Strongly Agree” to “Strongly Disagree” to the statement “The H1N1 vaccination campaign illustrated that our practice or pharmacy branch is capable of responding to large scale public health events.” We assessed participation in training drills or preparedness exercises by comparing practices responding “Yes” compared to those responding “No” or “Not sure”.

### Analysis

We used Fisher exact test to evaluate the bivariate association between each predictor and the outcome. We used logistic regression modeling to assess the effect of each predictor on response status and response timing. Models of best fit were determined based upon whether adding additional variables confounded the relationship between the primary variables of interest and the dependent variable by more than ten percent and whether they contributed significantly to R-squared. Only models containing miles to the nearest Target location and practice type were eligible for consideration as these were the primary variables of interest.

We used Fisher exact test to evaluate the association between late response (a proxy for non-response) and survey estimates to determine if late respondents answered differently than early respondents. We used late response as a proxy because we were interested in the impact of non-response bias on survey estimates, but we do not know how the non-responders would have answered. Late responders more closely resemble non-responders than early responders since, without follow-up, late responders would have likely been non-responders. For those factors that affected response status, we used logistic regression to assess the effects of each predictor on survey estimated effect measures of interest: ease of adherence to priority group vaccine guidelines, perceived capability to respond to future public health emergencies, and participation in training drills or preparedness exercises. If, for example, rural and metropolitan clinics adhere to guidelines similarly, then the finding that rural clinics are more likely to respond would not affect the quality of survey estimates. However, if rural clinics are more or less able to adhere to guidelines, we would want to determine the magnitude and direction of non-response bias on survey estimates.

All statistical analyses were performed using SAS v. 9.2 (Cary, NC). Results were considered statistically significant at an alpha level of 0.05 for all tests.

## Results

### Response Rate and Non-Response Bias

**Figure 1:**
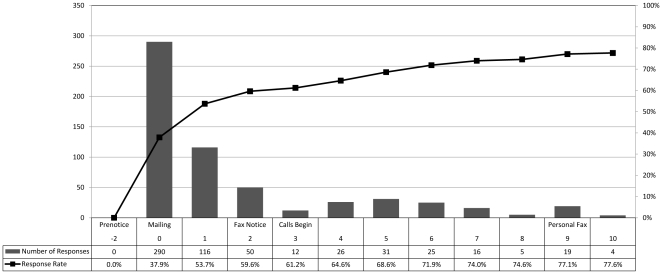
Number of responses and response rate by week and timing of follow-up.

Completed questionnaires were returned by 619 out of 765 (80.9%) vaccine providers sampled. Of these, 25 did not provide identifying information and could not be matched to the sample list. Since these providers could not be matched to demographic and geographic variables, these responses were included as non-respondents in this analysis. Of the 594 responses with identifying information and a valid time stamp, 404 (69.2%) were returned before any reminders were received. Number of responses and cumulative response rate by timing of follow-up are shown in Figure 1. Descriptive statistics are presented in Table 1.

Bivariate results stratified by response status and response timing are presented in Table 1. There was no significant difference between respondents and non-respondents by distance to Target store in miles (p = 0.730) or in minutes (p = 0.955). There was a significant difference among respondents and non-respondents by whether the practice regularly administers vaccines and by type of practice (Table 1). Those practices that regularly administer vaccines were significantly more likely to respond to the survey than those practices that provided H1N1 influenza vaccine only (82.4% compared to 74.6%, p = 0.013). By type of practice, response rates were lowest for pharmacies (69.4%) and highest for pediatric practices (91.4%) (p = 0.002).

Bivariate results stratified by early versus late response are also presented in Table 1. There was no significant difference between early respondents and late respondents by distance to the nearest Target in miles (p = 0.215) or in minutes (p = 0.196). There was a significant difference between early respondents and late respondents by whether the practice regularly administers vaccines (Table 1). Those practices that provided H1N1 influenza vaccine only were significantly more likely to respond late than those practices that regularly administer vaccines (34.9% compared to 25.1%, p = 0.014). The proportion of pharmacies that responded late was significantly higher than the proportion of traditional family practices that responded late (44.0% compared to 27.7%, p = 0.047).

Table 2 presents the logistic regression odds ratios (OR), 95% confidence intervals (95% CI) and p-values for the relationship between practice characteristics and survey response. Adjusting for type of practice, vaccinating for only H1N1, region of Washington, and degree of urbanicity, practices that were further from their nearest Target store were less likely to respond to the survey. The odds of receiving a response from a practice ten miles further from the nearest Target than another practice were 0.73 (95% CI = 0.60−0.89) times the odds of the nearer practice. Pediatric providers were significantly more likely than traditional family practice providers to respond to the survey (OR = 4.03, 95% CI = 1.36−11.96; Table 2). Non-traditional providers were significantly more likely to respond to the survey than traditional family practice providers (OR = 2.08, 95% CI = 1.06−4.08; Table 2). Providers that only provided H1N1 influenza vaccine were significantly less likely to respond than providers that regularly administer vaccines (OR = 0.56, 95% CI = 0.32−0.97; Table 2). Small town or rural providers were significantly more likely to respond to the survey than metropolitan providers (OR = 7.68, 95% CI = 1.44−40.88; Table 2).

**Table 2:** Logistic Regression: Association between response and timing of response with geographic and demographic variables.

		Response								Late Response							
		(vs. No Response)								(vs. Early Response)							
	Variable	Odds Ratio	95% Confidence Limits					*p*		Odds Ratio	95% Confidence Limits					*p*	
Miles to the nearest Target*		0.73	(	0.60	–	0.89	)	0.002	**	1.14	(	0.95	–	1.38	)	0.167	
Type of Practice (vs. Family Practice)																	
	Non-Traditional Vaccinators	2.08	(	1.06	–	4.08	)	0.033	**	0.79	(	0.39	–	1.62	)	0.526	
	Pediatric Providers	4.03	(	1.36	–	11.96	)	0.012	**	0.75	(	0.33	–	1.71	)	0.495	
	Pharmacy Providers	1.21	(	0.63	–	2.32	)	0.572		1.39	(	0.68	–	2.83	)	0.366	
	Government Providers	2.22	(	0.96	–	5.15	)	0.063		1.00	(	0.47	–	2.13	)	0.996	
	Hospital Providers	2.83	(	0.96	–	8.36	)	0.060		0.81	(	0.29	–	2.26	)	0.692	
	Corrections Facilities	2.30	(	0.79	–	6.69	)	0.125		1.16	(	0.44	–	3.10	)	0.765	
	Women's Health Providers	1.79	(	0.93	–	3.46	)	0.082		0.60	(	0.29	–	1.22	)	0.158	
Vaccinator for only H1N1																	
	(vs. vaccinator for more than H1N1)	0.56	(	0.32	–	0.97	)	0.040	**	1.73	(	0.99	–	3.04	)	0.056	
Region of Washington (vs. North)																	
	Northwest	1.90	(	0.69	–	5.26	)	0.215		0.62	(	0.25	–	1.58	)	0.317	
	West	0.89	(	0.41	–	1.96	)	0.755		1.12	(	0.52	–	2.43	)	0.767	
	Southwest	1.28	(	0.54	–	3.03	)	0.580		0.46	(	0.18	–	1.13	)	0.089	
	Tacoma	0.72	(	0.37	–	1.41	)	0.337		0.55	(	0.26	–	1.15	)	0.114	
	Seattle	0.96	(	0.52	–	1.74	)	0.885		0.75	(	0.40	–	1.39	)	0.355	
	North Central	1.04	(	0.33	–	3.31	)	0.946		1.15	(	0.38	–	3.42	)	0.808	
	South Central	2.25	(	0.87	–	5.79	)	0.094		1.15	(	0.55	–	2.42	)	0.711	
	East	0.76	(	0.36	–	1.58	)	0.457		0.83	(	0.38	–	1.82	)	0.646	
Urbanicity (vs. metropolitan)																	
	Micropolitan	2.74	(	1.00	–	7.54	)	0.051		0.71	(	0.30	–	1.69	)	0.441	
	Small Town or Rural	7.68	(	1.44	–	40.88	)	0.017	**	0.48	(	0.12	–	1.94	)	0.304	

Note: the model with late response as the dependent variable is not significant P>0.05.

*Per ten mile increase.

**P<0.05.

The logistic regression model predicting response status based on the set of predictors (distance to nearest Target store, type of practice, vaccinator for only H1N1, region of Washington, and degree of urbanicity) was significant (p<0.05), but the model R-squared was low (0.082). The self-reported predictors of practice size and VFC enrollment were not significantly associated with response status, and were not included in the final logistic regression model. Regression coefficients were calculated using both distance in miles and minutes to the nearest Target; using one or the other strategy gave similar regression coefficients and identical conclusions. The final model used distance in miles because its R-squared was slightly higher than the model using distance in minutes.

The logistic regression model comparing early respondents versus late respondents is presented in Table 2. There was no significant association between the set of predictors (distance to the nearest Target store, type of practice, vaccinator for only H1N1, region of Washington, degree of urbanicity) and late response (p  = 0.064, Table 2). Self-reported predictors practice size and VFC enrollment were not significantly associated with response status, and were not included in the final logistic regression model.

### Non-Response Bias and Survey Estimates

Next, we assessed the consequences of non-response bias in terms of demographic variables on the survey variables of interest: easy adherence to guidelines on priority groups, capability of the practice to respond to future public health emergencies, and whether the practice participated in disaster training or preparedness exercises.

There was no significant association between response timing (early/late) and key survey responses (Table 3). Table 4 presents logistic regression results predicting three survey estimates of interest based on the set of predictors shown to affect response (distance to nearest Target store in miles, type of practice, administering vaccinations for only H1N1, region of Washington, and degree of urbanicity). The model for ease of adherence to guidelines was not significant (p = 0.061). None of the models had a high R-squared, although R-squared was greater for the model predicting training or preparedness activities (R-squared = 0.254) compared to the models for ease of adherence to guidelines (R-squared = 0.067) and perceived practice capability to respond to public health emergencies (R-squared = 0.104). Each of the models for the survey estimates were influenced by different sets of independent variables. None of the independent variables were significant in more than one of the models predicting survey estimates.

**Table 3:** Survey estimates by timing of response.

	Total Respondents		Early Respondents		Late Respondents		P-Value*
Variable	N	%	N	%	N	%	
Adherence to guidelines on priority groups was easy (n = 569)	361	(62.9)	254	(64.5)	107	(61.1)	0.452
Practice is capable to respond to future public health emergencies (n = 567)	460	(81.1)	323	(82.2)	137	(78.7)	0.353
Participation in disaster training or preparedness exercises (n = 577)	253	(43.9)	186	(46.4)	67	(38.1)	0.069

Note: n varies by variable due to item specific non-response.

*P-values reported are Fisher exact tests between timing of response and survey answers.

**Table 4:** Logistic Regression: Association between survey estimates with geographic and demographic variables.

		Easy Adherence to Guidelines							Capable to Respond to Public Health Emergencies							Participation in disaster training or preparedness exercises						
		(vs. Moderate or Hard Difficulty)							(vs. Neutral or Not Capable)							(vs. No Training)						
Variable		Odds Ratio	95% Confidence Limits					*p*	Odds Ratio	95% Confidence Limits					*p*	Odds Ratio	95% Confidence Limits					*p*
Miles to the nearest Target *		0.87	(	0.72	–	1.05	)	0.157	0.87	(	0.65	–	1.16	)	0.342	1.15	(	0.92	–	1.43	)	0.232
Type of Practice (vs. Family Practice)																						
	Non-Traditional Vaccinators	1.31	(	0.65	–	2.62	)	0.455	0.71	(	0.31	–	1.61	)	0.402	1.36	(	0.68	–	2.73	)	0.389
	Pediatric Providers	1.65	(	0.79	–	3.48	)	0.187	2.10	(	0.66	–	6.64	)	0.208	1.71	(	0.82	–	3.55	)	0.154
	Pharmacy Providers	0.81	(	0.41	–	1.63	)	0.560	1.48	(	0.61	–	3.62	)	0.384	0.34	(	0.16	–	0.74	)	0.006
	Government Providers	0.82	(	0.41	–	1.65	)	0.581	0.74	(	0.30	–	1.90	)	0.513	3.30	(	1.54	–	7.06	)	0.002
	Hospital Providers	1.95	(	0.71	–	5.33	)	0.194	0.47	(	0.16	–	1.43	)	0.180	10.16	(	2.73	–	37.82	)	0.001
	Corrections Providers	0.74	(	0.27	–	1.98	)	0.541	1.81	(	0.45	–	7.59	)	0.407	7.32	(	2.16	–	24.80	)	0.001
	Women's Health Providers	1.61	(	0.83	–	3.12	)	0.155	0.93	(	0.42	–	2.08	)	0.853	0.74	(	0.38	–	1.44	)	0.375
Vaccinator for only H1N1																						
	(vs. vaccinator for more than H1N1)	1.34	(	0.78	–	2.29	)	0.288	0.77	(	0.40	–	1.46	)	0.430	1.17	(	0.67	–	2.04	)	0.587
Region of Washington (vs. North)																						
	Northwest	1.68	(	0.68	–	4.14	)	0.260	0.36	(	0.13	–	0.97	)	0.052	0.41	(	0.16	–	1.04	)	0.061
	West	1.73	(	0.78	–	3.82	)	0.178	1.15	(	0.38	–	3.48	)	0.802	0.69	(	0.30	–	1.55	)	0.366
	Southwest	1.47	(	0.64	–	3.35	)	0.360	0.55	(	0.20	–	1.48	)	0.238	1.56	(	0.67	–	3.62	)	0.303
	Tacoma	1.30	(	0.64	–	2.64	)	0.471	1.37	(	0.52	–	3.63	)	0.528	0.89	(	0.43	–	1.84	)	0.753
	Seattle	0.91	(	0.51	–	1.65	)	0.766	0.78	(	0.35	–	1.74	)	0.549	0.81	(	0.43	–	1.51	)	0.503
	North Central	0.63	(	0.21	–	1.91	)	0.413	3.18	(	0.34	–	28.59	)	0.309	1.09	(	0.32	–	3.71	)	0.888
	South Central	0.89	(	0.43	–	1.85	)	0.754	0.44	(	0.18	–	1.10	)	0.078	0.41	(	0.18	–	0.93	)	0.032
	East	0.77	(	0.37	–	1.64	)	0.503	0.72	(	0.26	–	2.00	)	0.518	0.29	(	0.12	–	0.70	)	0.006
Urbanicity (vs. metropolitan)																						
	Micropolitan	1.23	(	0.51	–	2.95	)	0.651	1.81	(	0.49	–	6.15	)	0.376	1.61	(	0.61	–	4.24	)	0.340
	Small Town or Rural	1.38	(	0.36	–	5.37	)	0.639	20.83	(	1.02	–	425.51	)	0.049	1.99	(	0.47	–	8.33	)	0.348

Note: model with Easy adherence as the dependent variable was not significant P>0.05.

*Per ten mile increase.

There was no significant observed effect of the number of miles to the nearest Target store on self-reported ease of adherence to guidelines (OR = 0.87, 95% CI = 0.72−1.05), perceived capability to respond to future public health emergencies (OR = 0.87, 95% CI = 0.65−1.16), or participation in disaster training or preparedness exercises (OR = 1.15, 95% CI = 0.92−1.43). Small town or rural location of the practice was positively associated with perceived capability to respond to future public health emergencies (OR = 20.83, 95% CI = 1.02−425.51). Practices located in small town or rural locations comprised 5.4% of sampled practices (41 of 765, Table 1) which accounts for the wide confidence intervals. The other variables shown to affect response (vaccination for only H1N1, and pediatric and non-traditional vaccinator provider types) were not significant in all models.

Our estimate of overall perceived capability of respond to public health emergencies is likely biased up and away from the null, because small town or rural location of the practice was positively associated with response and was also associated with increased perceived capability to respond to future public health emergencies. The magnitude of the bias is small. No other estimates associated with response were also associated with survey estimates; there is no evidence that survey estimates of ease of adherence to guidelines or participation in training exercises or preparedness drills were biased due to non-response.

## Discussion

This study supports previous study findings showing that incentives and study design factors improve response rates. We achieved a high response rate for this survey of Washington vaccine providers, which may have reduced potential non-response bias. However, we found statistically significant differences between respondents and non-respondents for four study variables – distance to the nearest Target, type of medical practice, whether the practice routinely administered more vaccines than H1N1, and urbanicity. Of particular interest, the negative association between distance to the nearest Target and response was significant and meaningfully large. This suggests that, while gift card incentives – along with other study design factors – can increase response rates, investigators should be aware that where and how gift cards can be used may affect who responds to the survey. If researchers use gift card incentives, they should use gift cards that appeal to and are easily redeemable by their study population. Even in a large state such as Washington, we saw that even relatively short distance increases to a Target location had a significant and meaningful impact on the probability of response.

However, those variables associated with non-response were not significantly related to survey estimates of interest, with the exception of small town or rural location of the practice. Small town or rural location was positively associated with response and with perceived capability to respond to future public health emergencies. This indicates that the overall survey estimate of perceived capability to respond to future public health emergencies is an overestimate. However, small town or rural practices made up a small fraction (5.4%, Table 1) of the sample, which suggests that non-response bias in this survey estimate is small. All other variables significantly associated with key survey estimates of interest were not significantly associated with response or timing of response.

The design of our study built on existing work evaluating methods to increase response rates. We used multiple evidence-based methods to ensure a high response rate for our survey, and thus we are unable to evaluate the effectiveness of individual protocol design factors. As pointed out in previous studies, it is important to have a broad set of demographic and practice variables available on the entire sampling frame [8]. In our study, the variables available on the entire sampling frame explained only a small proportion of the variation in response, timing of response, and survey estimates. Low model R-squared indicates that there are likely several unmeasured factors associated with survey response. Variables such as the role of the survey contact, number of patients vaccinated, and respondent income were not considered for analysis because these variables were not available for non-responders. Although some variables were associated with response and survey estimates, almost 20% of sampled H1N1 vaccine providers did not respond and we do not know how they would have answered.

Further research could explore the relationship between distance to the nearest place to redeem gift cards and likelihood of response. This should include surveys that are specifically designed to compare gift cards to cash incentives, take place outside of Washington, or provide gift cards to stores other than Target to assess whether results are context-specific.

Future surveys using gift card incentives would benefit from making an informed choice about gift card selection. Investigators should consider the geographic distribution of the selected gift card store, the option of redeeming the gift card online, and internet access among respondents during project planning.
